# Effects of single-anastomosis duodenal–ileal bypass with sleeve gastrectomy on gut microbiota and glucose metabolism in rats with type 2 diabetes

**DOI:** 10.3389/fmicb.2024.1357749

**Published:** 2024-05-27

**Authors:** Lun Wang, Shixing Li, Tao Jiang

**Affiliations:** ^1^Department of Gastrointestinal Surgery, Affiliated Hospital of Zunyi Medical University, Zunyi, China; ^2^Department of Bariatric and Metabolic Surgery, China-Japan Union Hospital of Jilin University, Changchun, China

**Keywords:** single-anastomosis duodenal–ileal bypass with sleeve gastrectomy, type 2 diabetes, gut microbiota, bariatric and metabolic surgery, SADI-S, T2D

## Abstract

**Background:**

Bariatric and metabolic surgery often leads to significant changes in gut microbiota composition, indicating that changes in gut microbiota after bariatric and metabolic surgery might play a role in ameliorating type 2 diabetes (T2D). However, the effects of single-anastomosis duodenal–ileal bypass with sleeve gastrectomy (SADI-S) on gut microbiota in T2D remain unclear.

**Objectives:**

To investigate the effects of SADI-S on gut microbiota and glucose metabolism in T2D rats.

**Methods:**

Nineteen T2D rats were randomly divided into the SADI-S group (*n* = 10) and the sham operation with pair-feeding group (sham-PF, *n* = 9). Fecal samples were collected to analyze the gut microbiota composition with 16S ribosomal DNA gene sequencing. The fasting blood glucose and glycated hemoglobin were measured to evaluate the effects of SADI-S on glucose metabolism.

**Results:**

The Chao and ACE index results indicated the richness of the gut microbial community. The ACE and Chao index values were significantly lower in the SADI-S group than in the sham-PF group, indicating that indicating that species richness was significantly lower in the SADI-S group than in the sham-PF group (*p* < 0.05). Shannon and Simpson indices were used to estimate the species diversity of the gut microbiota. Compared with the sham-PF group, the SADI-S group showed significantly lower Shannon index and higher Simpson index values, indicating that the species diversity was significantly lower in the SADI-S group than in the sham-PF group (*p* < 0.05). At the genus level, SADI-S significantly changed the abundances of 33 bacteria, including the increased anti-inflammatory bacteria (*Akkermansia* and *Bifidobacterium*) and decreased pro-inflammatory bacteria (*Bacteroides*). SADI-S significantly decreased the fasting blood glucose and glycated hemoglobin levels. The blood glucose level of rats was positively correlated with the relative abundances of 12 bacteria, including *Bacteroides*, and negatively correlated with the relative abundances of seven bacteria, including *Bifidobacterium*.

**Conclusion:**

SADI-S significantly altered the gut microbiota composition of T2D rats, including the increased anti-inflammatory bacteria (*Akkermansia* and *Bifidobacterium*) and decreased pro-inflammatory bacteria (Bacteroides). The blood glucose level of rats was positively correlated with the abundances of 12 bacteria, including *Bacteroides*, but negatively correlated with the relative abundance of 7 bacteria, including *Bifidobacterium*. These alternations in gut microbiota may be the mechanism through which SADI-S improved T2D. More studies should be performed in the future to validate these effects.

## Introduction

Type 2 diabetes (T2D) is a chronic endocrine disease characterized by insulin resistance and/or decreased insulin secretion. According to the International Diabetes Federation (2021), the global adult population (age: 20–79 years) with diabetes was 537 million in 2021, with T2D accounting for approximately 90% of cases. Thus, diabetes leads to a huge financial burden of health expenditure and seriously threatens people’s lives. Bariatric and metabolic surgery has demonstrated a more prolonged effect than conventional therapeutic strategies in treating T2D (Schauer et al., 2012; Kashyap et al., 2013; Mingrone et al., 2021). Currently, sleeve gastrectomy (SG), Roux-en-Y gastric bypass (RYGB), biliopancreatic diversion with duodenal switch (BPD/DS), and single-anastomosis duodenal–ileal bypass with sleeve gastrectomy (SADI-S) are routinely used to treat obesity and T2D. Among them, SG and RYGB are the most commonly performed bariatric surgeries, while BPD/DS is the least common because of potential malnutrition risks (Angrisani et al., 2021). Similar to BPD/DS, SADI-S is able to provide weight loss and diabetes remission. Additionally, it reduces the operative and malnutrition risks compared to BPD/DS by reducing an anastomotic stoma and lengthening the common channel in the small intestine (Sánchez-Pernaute et al., 2007).

Gut microbiota and its-derived metabolites participate in the initiation and progression of T2D through distinct signaling pathways, mainly including short-chain fatty acids (SCFA), bile acid, branched-chain amino acids (BCAA), and lipopolysaccharide (LPS). SCFA is a metabolite produced by intestinal bacteria to metabolize dietary fiber, including acetate, propionate acid, and butyrate. SCFA can stimulate the secretion of peptide YY (PYY) and glucagon-like peptide-1 (GLP-1) by colon L cells, which are responsible for delaying gastric emptying, inhibiting appetite, promoting insulin secretion, and reducing glucagon, thereby affecting the development of diabetes (Lin et al., 2012; Tolhurst et al., 2012; Kaji et al., 2014; Psichas et al., 2015). Using bidirectional Mendelian randomization (MR) analyses to assess the causality between microbial characteristics and blood glucose characteristics, Sanna et al. (2019) found that the increase of butyrate driven by host genetics was related to the improvement of insulin response after the oral glucose tolerance test. Primary bile acids are converted into secondary bile acids by gut microbiota, affecting glucose metabolism and insulin sensitivity through different signaling pathways (Fiorucci and Distrutti, 2015). Secondary bile acid stimulates the farnesoid X receptor (FXR) and leads to the release of fibroblast growth factor 19/15 (FGF19/15). FGF19/15 acts as ligands to improve insulin sensitivity and glucose tolerance. Secondary bile acid can also activate the thiol guanosine receptor-5 (TGR-5) receptor, promote muscle energy consumption and the secretion of GLP-1 by intestinal L cells, and rescue insulin resistance and abnormal glucose metabolism (Shapiro et al., 2018; Zheng et al., 2021). BCAA include valine, leucine, and isoleucine. They are essential amino acids for the human body, and cannot be synthesized by the host, must be obtained from diet, and are mainly produced by gut microbiota metabolism (Katsanos et al., 2006). Increased intake of BCAA in diet promotes the development of T2D and insulin resistance (Zheng et al., 2016; Asghari et al., 2018). The main driving bacteria for the synthesis of BCAA are Prevotella and Bacteroides, feces microbiota transplantation of Prevotella in a mouse model can induce insulin resistance, aggravate glucose intolerance and augment circulating levels of BCAA (Pedersen et al., 2016). The mechanism of insulin resistance induced by BCAA is found to be closely related to the mTOR signaling pathway. High expression of phosphorylated mTORSer2448, phosphorylated S6K1Thr389, and phosphorylated IRS1Ser302 has been found in mice fed with BCAA, which can block the normal conduction of insulin signaling and cause insulin resistance (Newgard et al., 2009). A number of studies have shown that T2D patients have a low degree of inflammation due to increased LPS in the peripheral circulation (Pussinen et al., 2011; Jayashree et al., 2014; Gomes et al., 2017). LPS recognizes the receptor TLR4 with the help of CD14, which leads to macrophage aggregation and NF-κB inflammatory signaling pathway activation. After that, abnormal phosphorylation of insulin receptor substrate and insulin resistance occur (Creely et al., 2007).

The remarkable glycaemic improvement after bariatric surgery occurred concomitantly with changes in the gut microbiota. More importantly, symptoms of diabetic patients can be improved by modifying gut microbiota. Murphy et al. (2017) found that only an increase in *Roseburia* species was observed among those achieving diabetes remission after RYGB and SG, this species has been observed to increase after fecal microbiota transplantation from lean to obese donors together with improved insulin sensitivity (Vrieze et al., 2012) and to be decreased in T2D (Qin et al., 2012; Karlsson et al., 2013). By combining clinical and preclinical studies, Debédat et al. (2022) demonstrated that the severity of T2D is associated with an enrichment of the class Bacteroidia both before and after RYGB, transitions of T2D severity due to important metabolic improvements after RYGB are associated with a strong decrease in Bacteroidia. Anhê et al. (2023) demonstrated that exposure of rodents to human gut microbiota after bariatric surgery improves glycaemic control. Despite emerging metabolic studies on bariatric and metabolic surgery, the effects of SADI-S on gut microbiota remain unknown. The aim of the present study was to explore the effects of SADI-S on gut microbiota and glucose metabolism of T2D rats.

## Materials and methods

### Establishment of a T2D rat model and experimental design

The study protocol was approved by the Animal Experiment Ethics Committee of the First Hospital of Jilin University. The rats were fed according to the national guidelines for the care of animals in the People’s Republic of China. Twenty male Wistar rats (8 weeks) were purchased from the Vital River Laboratory Animal Technology Co., Ltd. (Beijing, China). They were housed in independent cages at constant temperature and humidity, with free access to food and water and a 12-h light/12-h dark cycle. After 2 weeks of adaptive feeding with an ordinary diet (containing 13.8% fat, 63.4% carbohydrate, and 22.8% protein), the rats were switched to a high-fat diet (containing 45.6% fat, 37.9% carbohydrate, and 16.5% protein). After 8 weeks of high-fat feeding, the T2D model was established according to a protocol. First, the rats were fasted for 12 h and weighed. Second, streptozotocin (STZ; Sigma, USA) was dissolved in an ice-cold citrate sodium buffer (0.1 mol/L, pH = 4.5) to prepare an STZ solution with a concentration of 10 mg/ml. Third, 30 mg/kg of STZ was injected intraperitoneally.

The T2D rat model was considered to be successful when rats’ random blood glucose level was at least 16.7 mmol/L 72 h after the STZ injection. During the first 5 days after the STZ injection, Novolin^®^ N was injected subcutaneously to control the rats’ blood glucose level to avoid death from hyperglycemia. Due to the blood glucose level below 16.7 mmol/L, one rat was injected with STZ at a dose of 30 mg/kg again after its blood glucose dropped to the normal range. Unfortunately, the blood glucose in the first, second and third day after the second STZ injection was 9.1, 11.3, and 7.1 mmol/L, respectively. Consequently, this rat was finally defined as failure of establishing model. Therefore, 19 rat models of T2D were successfully established, which were divided into the SADI-S group (*n* = 10) and the sham operation with pair-feeding group (*n* = 9). To diminish the potential impact of food difference between groups on experimental results. The SADI-S group and sham-PF group were pair-fed to maintain the same kind of food and the same amount of food intake in the postoperative period.

### Preoperative preparation

The night before surgery, the rats were placed in a cage with raised wire platforms to prevent coprophagy and fasted for 12 h. Before anesthesia, the rats’ blood glucose level was routinely measured. When the blood glucose level was too high, an appropriate dose of insulin was injected to decrease the surgical risk from hyperglycemia. We dissolved 1 g of pentobarbital sodium in 100 ml of double-distilled water to prepare a solution of sodium pentobarbital with a concentration of 10 mg/ml and stored it in a sterile light-proof bottle.

The rats’ abdominal fur was shaved from the xiphoid to the groin using an electric hair clipper. Parts of the dorsal and femoral fur were also shaved to facilitate postoperative hydration and injections of antibacterial agents. When the blood glucose level was confirmed to not be too high, pentobarbital sodium was used for anesthesia by peritoneal injection at a dose of 35 mg/kg. Subsequently, 10 ml of 0.9% saline was injected at multiple sites on the back. We placed the rats on the operating table in the supine position and fixed their limbs. Subsequently, we disinfected their abdominal skin with iodophor and covered it with a sterile surgical sheet.

### Surgical procedure and postoperative care

The protocols of SADI-S and postoperative care was performed according to our previous study (Wang et al., 2022).

### Intraperitoneal glucose tolerance test

Intraperitoneal glucose tolerance test (IPGTT) was performed preoperatively and 8 weeks postoperatively. After fasting for 12 h, the rats were intraperitoneally injected with 2 g/kg of 50% glucose. We measured the blood glucose level by pricking the tail vein before and 15, 30, 60, 120, and 180 min after injecting glucose. Finally, we plotted the blood glucose–time curve and calculated the area under the curve (AUC) of the glucose level in IPGTT using the following formula: AUC = (G0 + G15) × 15/2 + (G15 + G30) × 15/2 + (G30 + G60) × 30/2 + (G60 + G120) × 60/2 + (G120 + G180) × 60/2, wherein G was the blood glucose value before and 15, 30, 60, 120, and 180 min after injecting glucose.

### Histological assessment of pancreatic tissues

At 8 weeks postoperatively, the rats’ pancreatic tissue samples were fixed in the paraformaldehyde solution, embedded, cut into 4-μm sections, stained with a double-labeling immunofluorescence stain, and observed under a light microscope for pathological changes.

### Sample collection and storing

At 8 weeks postoperatively, fecal samples were collected and immediately frozen in liquid nitrogen, and stored at −80°C until further gut microbiota assays.

### Sequencing and analysis of gut microbiota

#### Sample DNA extraction and targeted amplification and sequencing

Genomic DNA was extracted according to the protocol of the QIAamp DNA Stool Mini Kit (Qiagen, Hilden, Germany). The V3–V4 region of the bacterial 16S ribosomal DNA gene was amplified with a polymerase chain reaction (PCR) using the barcoded primers 357F 5′-ACTCCTACGGRAG GCAG C AG-3′ and 806R 5′-GGACTACHVGGGTWTCTAAT-3′. The PCR system contained 10 μl of 5× buffer, 1 μl of 10 mM deoxynucleoside triphosphate, 1 U of Phusion Ultra High-Fidelity DNA Polymerase, 1 μl each of 10 μM forward and reverse primers, and 20–50 ng of template DNA, followed by supplementation with 50 μl of ultrapure water. PCR conditions were: 94°C for 2 min, followed by 24 cycles at 94°C for 30 s, 56°C for 30 s, 72°C for 30 s, and 72°C for 5 min. PCR products were recycled using the AxyPrep DNA Gel Extraction Kit (Axygen Scientific, Inc., USA) and quantified using the FTC-3000™ Real-Time PCR Cycler (Fengling, Shanghai, China). With an equal molar mixing ratio, we repeated PCR amplification using the following products: 8 μl of 5× buffer, 1 μl of 10 mM deoxynucleoside triphosphate, 0.8 U of Phusion Ultra High-fidelity DNA Polymerase, 1 μl each of 10 μM forward and reverse primers, and 5 μl of template DNA, followed by supplementation with 40 μl of ultrapure water. PCR conditions were: 94°C for 2 min, followed by eight cycles at 94°C for 30 s, 56°C for 30 s, 72°C for 30 s, 72°C for 5 min, and incubation at 10°C. The adapters, primers, and barcodes required for sequencing in the Illumina platform were added to both ends of the targeted fragments to complete the library construction. The constructed library was sequenced using the NovaSeq 6000 SP 500 Cycle Reagent Kit (Illumina, USA).

#### Data processing and analysis

After acquiring raw data, reads were assigned to each sample using a barcode to obtain a valid sequence. Low-quality sequences at the ends of sequencing results were removed using Trimmomatic software version 0.35. Sequencing adapters and primers were processed using the Cutadapt software (version 1.16). Based on the overlap relationship between pair-end reads, paired reads were spliced into a sequence using Flash software version 1.2.11. To obtain optimized sequences, low-quality sequences were removed using Mothur software (version 1.33.3) with the following parameters: maxambig = 0; maxhomop = 8; minlength = 200; and maxlength = 485. Subsequently, operational taxonomic units (OTUs) were clustered with 97% similarity cutoffs using UPARSE software usearch (version 8.1.1756).^1^ Representative OTUs were aligned with the SILVA 128 database to annotate species information. Based on the taxonomic information, the community structure was analyzed at levels of phylum, class, order, family, genus, and species and visualized using the “ggplot2” package (version 3.3.3) of R language (version 3.6.3). The alpha diversity analysis, including Chao, ACE, Shannon, and Simpson indices, was performed using the “vegan” package of R language version 3.6.3 to estimate fecal microbial species richness and diversity. Based on the evolutionary distance of sequences and the abundance of OTUs, beta diversity was performed using weighted.unifrac and then displayed by NMDS. The statistical significance of similarity to reflect the differences of gut microbiota composition between the SADI-S group and Sham-PF group was tested using the analysis of similarities (Anosim). In addition, the rarefaction curve was used to judge whether or not the amount of sequencing data was reasonable. The species accumulation curve was used to judge whether or not the sample size was sufficient. The rank–abundance curve was used to determine species abundance and evenness. The principal component analysis was performed to reflect differences and distances between samples.

### Statistical analysis

Statistical analyses were performed using SPSS 22.0, GraphPad Prism 8.0.2, and R version 3.6.3. The Wilcoxon rank sum test or independent sample *t*-test was performed to compare the differences between the two groups. Spearman correlation analysis was performed to evaluate the correlation between metabolic indices and gut microbiota. A *p*-value < 0.05 was considered to indicate statistical significance.

## Results

### Surgical outcomes

In the SADI-S group, four rats died postoperatively, three of which died from bleeding while one from anastomotic leakage, and six rats survived for 8 weeks postoperatively until they were sacrificed. In the sham-PF group, one rat died from incision rupture on postoperative day 12, and eight rats survived for 8 weeks postoperatively until they were sacrificed.

### Changes in the body weight and the glucose metabolism

**FIGURE 1:**
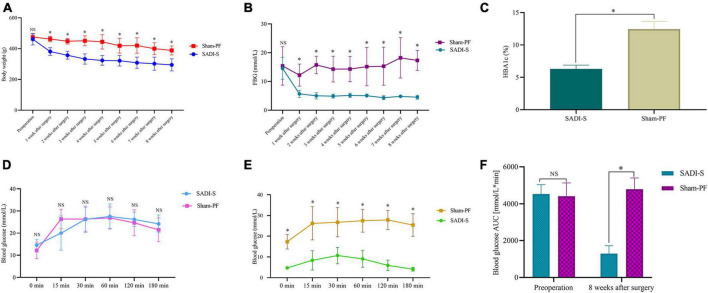
Changes in the body weight **(A)**, fasting blood glucose level **(B)**, glycated hemoglobin level **(C)**, and area under the curve of the glucose level in the intraperitoneal glucose tolerance test (IPGTT) **(F)** preoperatively and postoperatively. Changes in the blood glucose level in IPGTT at each time point preoperatively **(D)** and 8 weeks postoperatively **(E)**. *Significant difference (*p* < 0.05); NS, no significance (*p* > 0.05). HBA1c, glycated hemoglobin; AUC, area under the curve; IPGTT, intraperitoneal glucose tolerance test.

Figure 1A shows the changes in the body weight. The body weight was significantly lower in the SADI-S group than in the sham-PF groups (*p* < 0.05). In addition, the body weight showed a downward trend postoperatively in the sham-PF groups. However, the decrease in the body weight at each postoperative time point was significantly greater in the SADI-S group than in the sham-PF groups (*p* < 0.05).

Fasting blood glucose (FBG) and glycated hemoglobin (HBA1c) levels were significantly lower in the SADI-S group than in the sham-PF groups (Figures 1B, C) (*p* < 0.05). Figures 1D, E show changes in the blood glucose level in IPGTT at each time point preoperatively and postoperatively. Preoperative blood glucose levels in IPGTT at any time point did not differ between the two groups (*p* > 0.05; Figure 1D). However, 8-week postoperative blood glucose levels in IPGTT at all time points were significantly lower in the SADI-S group than in the sham-PF group (*p* < 0.05; Figure 1E). Figure 1F shows changes in AUC of the blood glucose level in IPGTT preoperatively and postoperatively. Preoperative changes in AUC of the blood glucose level in IPGTT did not differ significantly between the SADI-S group and sham-PF group (*p* > 0.05). However, 8-week postoperative changes in AUC of the blood glucose level in IPGTT was significantly lower in the SADI-S group than in the sham-PF groups (*p* < 0.05).

### Histological assessment of pancreatic tissues

**FIGURE 2:**
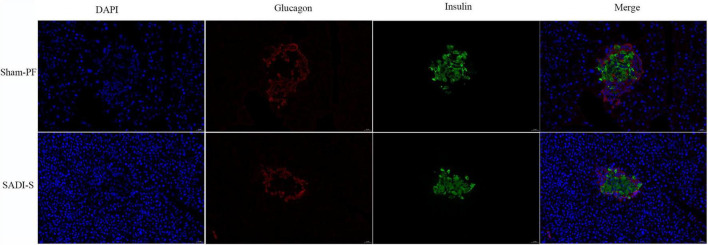
Double-labeling immunofluorescence images. Morphological and histological evaluations of pancreatic tissues in the two groups. The red color represents the pancreatic islet α-cells. The green color represents the pancreatic islet β-cells. The blue color represents the cell nucleus.

Figure 2 shows the double-labeling immunofluorescence results of pancreatic tissues. In the sham-PF group, the pancreatic islet β-cells were injured severely with marked vacuolation degeneration compared to the SADI-S group. Further, the pancreatic islet β- and α-cells were distributed disorderly. In contrast, the SADI-S group showed complete pancreatic islets and compact and uniform morphology of pancreatic islet β-cells without significant vacuolar degeneration compared to the sham-PF group.

### Rarefaction and species accumulation curves

**FIGURE 3:**
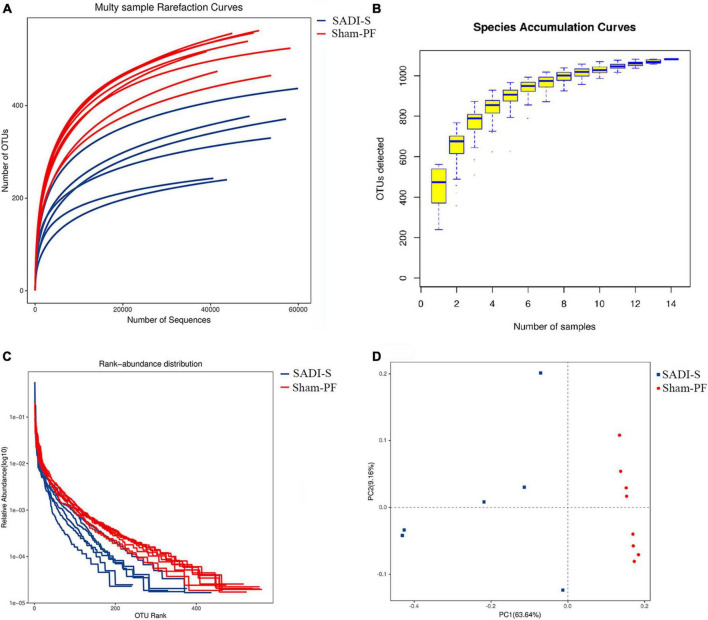
Evaluations of the rarefaction curve **(A)**, species accumulation curve **(B)**, rank–abundance curve **(C)**, and principal component **(D)** in the two groups 8 weeks postoperatively.

The rarefaction curve was used to judge whether or not the amount of sequencing data of the sample was reasonable. With increasing sequencing amount, the increasing trend of the number of OTUs gradually slowed down and reached saturation (Figure 3A), suggesting that the amount of sequencing could reflect the gut microbiota composition of rats. More data only generated a small amount of new OTUs. Thus, the amount of sequencing data used in this study was reasonable. In addition, the rarefaction curve was used to compare the species abundance in samples with different amounts of sequencing data. Bacterial abundance in the sham-PF group was significantly higher compared to the SADI-S group (Figure 3A).

The species accumulation curve was used to reflect changes in species abundance with increasing sample size, which is widely used to judge the adequacy of a sample size. The increasing trend in the OTUs slowed down with increasing sample size (Figure 3B), suggesting that species abundance would not increase significantly with increasing sample size in this environment. Thus, our sample size was sufficient and could be used for data analyses.

### Rank–abundance curve and principal component analysis

The rank–abundance curve was used to determine species abundance and evenness. The species abundance is reflected by the width of the curve: the greater the species abundance, the farther is the curve on the horizontal axis. The species evenness is reflected by the shape (smoothness) of the curve: The shallower the curve, the more even is the species distribution. The width of the curve in the horizontal axis was shorter in the SADI-S group than in the sham-PF group (Figure 3C), indicating that the species abundance was lower in the SADI-S group than in the sham-PF group. The shape of the curve was steeper in the SADI-S group than in the sham-PF group, suggesting that species evenness was lower in the SADI-S group than in the sham-PF group.

The principal component analysis was used to reflect the differences and distance between the samples. The SADI-S group was separated from the sham-PF groups in the direction of the first principal component axis (Figure 3D), suggesting that SADI-S was the main factor causing this separation.

### Changes in gut microbiota diversity

**FIGURE 4:**
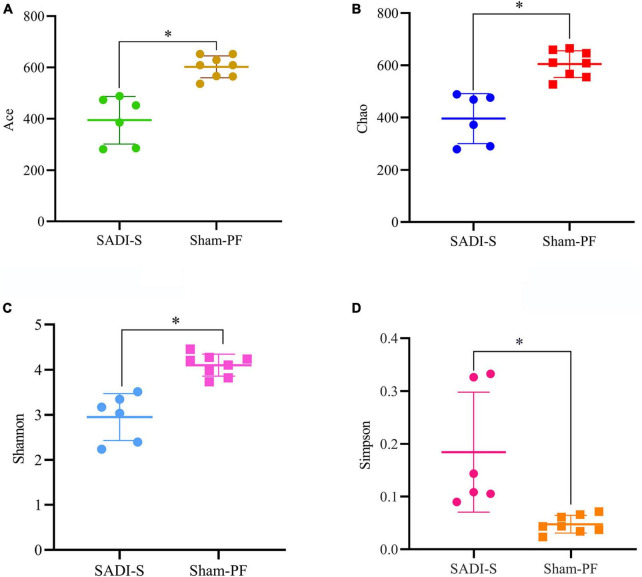
Comparison of the α-diversity of the gut microbiota. Changes in the ACE **(A)**, Chao **(B)**, Shannon **(C)**, and Simpson **(D)** indices in the two groups 8 weeks postoperatively.

A total of 690,893 optimized sequences were obtained from 14 samples, which were divided into 1,082 OTUs. A total of 606 common types of OTUs were found between the two groups; more unique types of OTUs were found in the sham-PF group than the SADI-S group (427 vs. 49) (Supplementary Figure 1). Good’s coverage index of each sample exceeded 99.7%, indicating that the sequencing amount of each sample reached saturation. Alpha diversity analysis (including Chao, Ace, Shannon, and Simpson) was performed to estimate the species richness and diversity of gut microbiota (Figure 4). The Chao and ACE index indicated the richness of gut microbial community. The ACE and Chao index in the SADI-S group were significantly lower than that of the sham-PF group, indicating that the species richness in the SADI-S group was significantly lower compared to the sham-PF group (*p* < 0.05). Shannon and Simpson indices were used to estimate species diversity of gut microbiota.

Shannon index reflects the uncertainty in predicting the species of the collected individual. The greater the Shannon index, the greater the uncertainty of prediction, indicating the higher the diversity of gut microbial community. In contrast, Simpson index reflects the probability that individuals obtained from a community species by two consecutive sampling belong to the same species. The greater the Simpson index, the greater the probability of acquiring the same species from two consecutive sampling, indicating the lower the species diversity of gut microbial community. A significantly decreased Shannon index and increased Simpson index were observed in the SADI-S group than that of the sham-PF group, indicating that the species diversity in the SADI-S group was significantly lower compared to the sham-PF groups (*p* < 0.05).

Based on the evolutionary distance of sequences and the abundance of OTUs, weighted unifrac was used for creating the distance matrix between samples and then Anosim was used to test for significance of similarity between samples. As shown in Supplementary Figure 2, the SADI-S group were clearly separated from the sham-PF group, indicating that the gut microbiota composition was significantly changed after SADI-S.

### Changes in the taxonomic composition of gut microbiota

**FIGURE 5:**
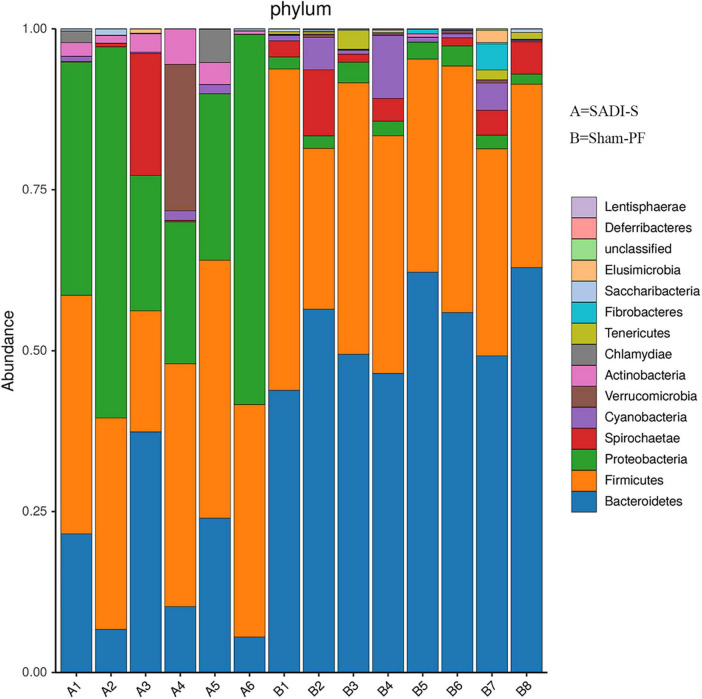
Changes in the taxonomic composition of the gut microbiota at the phylum level.

Based on the phylogenetic analysis, a total of 1,082 OTUs were obtained from 14 stool samples and belonged to 14 phyla (Figure 5). The 10 most abundant phyla were Bacteroidota, Firmicutes, Proteobacteria, Spirochaetota, Cyanobacteria, Verrucomicrobia, Actinomycetota, Chlamydiae, Tenericutes, and Fibrobacteres. Bacteroidota had the highest proportion (38.0%), followed by Firmicutes (34.9%), Proteobacteria (17.1%), Spirochaetota (3.4%), Cyanobacteria (1.9%), Verrucomicrobia (1.7%), and Actinobacteria (1.2%). The proportions for the remaining phyla were below 1%. The cladogram of plots presented the LEfSe results of the biological structure of gut microbiota (Supplementary Figure 3). Gut bacteria marked with small circles highlight significant differences in relative abundance between groups. The circles have different layers, which represent different taxonomic ranks (phylum, class, order, family, genus, and species, respectively) from inside to outside. The diameter of each circle is proportional to the abundance of the taxon. Each node (circle) represents a taxon: the blue nodes represent the enrichment of gut bacteria at 8 weeks post-surgery in the SADI-S group, the red nodes indicate a higher relative abundance in the sham-PF group. Yellow nodes account for those gut bacteria that were not statistically and biologically different in terms of abundance between groups.

Table 1 shows the results of abundance of gut microbiota with significant difference between the SADI-S group and sham-PF group. At the phylum level, the abundances of Bacteroidota, Tenericutes, and Fibrobacterota were significantly lower in the SADI-S group than in the sham-PF group, while those of Proteobacteria, Verrucomicrobiota, Actinomycetota, and Chlamydiota were significantly higher.

**TABLE 1:** The comparison of abundance of gut microbiota with significant difference between the SADI-S group and sham-PF group.

	SADI-S group	Sham-PF group	*p* Value
**Phylum level**			
Bacteroidetes	0.1759 ± 0.1237	0.5332 ± 0.0714	0.0007
Proteobacteria	0.3670 ± 0.1706	0.0233 ± 0.0059	0.0007
Verrucomicrobia	0.0381 ± 0.0930	0.0019 ± 0.0013	0.0426
Actinobacteria	0.0259 ± 0.0179	0.0017 ± 0.0014	0.0013
Chlamydiae	0.0115 ± 0.0207	0.0000 ± 0.0001	0.0187
Tenericutes	0.0002 ± 0.0002	0.0080 ± 0.0100	0.0127
Fibrobacteres	0.0000 ± 0.0000	0.0062 ± 0.0141	0.0124
**Class level**			
Bacteroidia	0.1756 ± 0.1237	0.5125 ± 0.0774	0.0007
Clostridia	0.1862 ± 0.0527	0.2885 ± 0.0960	0.0426
Gammaproteobacteria	0.3503 ± 0.1825	0.0027 ± 0.0026	0.0007
Erysipelotrichia	0.0460 ± 0.0496	0.0018 ± 0.0017	0.0127
Verrucomicrobiae	0.0381 ± 0.0930	0.0001 ± 0.0003	0.0146
Coriobacteriia	0.0140 ± 0.0176	0.0017 ± 0.0014	0.0293
Actinobacteria	0.0119 ± 0.0088	0.0000 ± 0.0000	0.0024
Chlamydia	0.0115 ± 0.0207	0.0000 ± 0.0001	0.0187
Mollicutes	0.0002 ± 0.0002	0.0080 ± 0.0100	0.0127
Fibrobacteria	0.0000 ± 0.0000	0.0062 ± 0.0141	0.0124
Betaproteobacteria	0.0053 ± 0.0067	0.0011 ± 0.0009	0.0293
Epsilonproteobacteria	0.0003 ± 0.0006	0.0040 ± 0.0032	0.0080
Opitutae	0.0000 ± 0.0000	0.0003 ± 0.0004	0.0291
**Order level**			
Bacteroidales	0.1756 ± 0.1237	0.5125 ± 0.0774	0.0007
Clostridiales	0.1862 ± 0.0527	0.2883 ± 0.0959	0.0426
Enterobacteriales	0.3481 ± 0.1828	0.0008 ± 0.0008	0.0007
Erysipelotrichales	0.0460 ± 0.0496	0.0018 ± 0.0017	0.0127
Verrucomicrobiales	0.0381 ± 0.0930	0.0001 ± 0.0003	0.0146
Coriobacteriales	0.0140 ± 0.0176	0.0017 ± 0.0014	0.0293
Bifidobacteriales	0.0116 ± 0.0090	0.0000 ± 0.0000	0.0016
Chlamydiales	0.0115 ± 0.0207	0.0000 ± 0.0001	0.0187
Fibrobacterales	0.0000 ± 0.0000	0.0062 ± 0.0141	0.0124
Burkholderiales	0.0053 ± 0.0067	0.0011 ± 0.0009	0.0293
Campylobacterales	0.0003 ± 0.0006	0.0040 ± 0.0032	0.0080
Thermoanaerobacterales	0.0000 ± 0.0000	0.0001 ± 0.0000	0.0124
**Family level**			
Prevotellaceae	0.0787 ± 0.0925	0.3954 ± 0.1345	0.0013
Enterobacteriaceae	0.3481 ± 0.1828	0.0008 ± 0.0008	0.0007
Ruminococcaceae	0.0765 ± 0.0225	0.1587 ± 0.0546	0.0007
Lactobacillaceae	0.0117 ± 0.0187	0.0437 ± 0.0303	0.0200
Erysipelotrichaceae	0.0460 ± 0.0496	0.0018 ± 0.0017	0.0127
Bacteroidaceae	0.0070 ± 0.0058	0.0259 ± 0.0128	0.0047
Verrucomicrobiaceae	0.0381 ± 0.0930	0.0001 ± 0.0003	0.0146
Rikenellaceae	0.0036 ± 0.0062	0.0224 ± 0.0161	0.0080
Streptococcaceae	0.0238 ± 0.0398	0.0003 ± 0.0003	0.0080
Coriobacteriaceae	0.0140 ± 0.0176	0.0017 ± 0.0014	0.0293
Bifidobacteriaceae	0.0116 ± 0.0090	0.0000 ± 0.0000	0.0016
Chlamydiaceae	0.0115 ± 0.0207	0.0000 ± 0.0001	0.0187
Porphyromonadaceae	0.0016 ± 0.0025	0.0060 ± 0.0033	0.0127
Fibrobacteraceae	0.0000 ± 0.0000	0.0062 ± 0.0141	0.0124
Alcaligenaceae	0.0052 ± 0.0067	0.0011 ± 0.0009	0.0293
Enterococcaceae	0.0059 ± 0.0063	0.0000 ± 0.0000	0.0019
Helicobacteraceae	0.0003 ± 0.0006	0.0040 ± 0.0032	0.0080
Clostridiaceae	0.0015 ± 0.0023	0.0001 ± 0.0001	0.0117
Eubacteriaceae	0.0015 ± 0.0017	0.0000 ± 0.0000	0.0019
Defluviitaleaceae	0.0001 ± 0.0001	0.0004 ± 0.0003	0.0115
Thermoanaerobacteraceae	0.0000 ± 0.0000	0.0001 ± 0.0000	0.0124
**Genera level**			
*Escherichia*	0.3459 ± 0.1805	0.0007 ± 0.0008	0.0016
*Prevotella*	0.0151 ± 0.0145	0.1135 ± 0.0659	0.0187
*Alloprevotella*	0.0163 ± 0.0221	0.0529 ± 0.0354	0.0007
*Lactobacillus*	0.0117 ± 0.0187	0.0437 ± 0.0303	0.0200
*Ruminococcus*	0.0046 ± 0.0067	0.0421 ± 0.0134	0.0027
*Bacteroides*	0.0070 ± 0.0058	0.0259 ± 0.0128	0.0007
*Akkermansia*	0.0381 ± 0.0930	0.0001 ± 0.0003	0.0124
*Streptococcus*	0.0238 ± 0.0398	0.0003 ± 0.0003	0.0080
*Quinella*	0.0026 ± 0.0054	0.0107 ± 0.0076	0.0019
*Bifidobacterium*	0.0116 ± 0.0090	0.0000 ± 0.0000	0.0080
*Chlamydia*	0.0115 ± 0.0207	0.0000 ± 0.0001	0.0293
*Anaerotruncus*	0.0017 ± 0.0011	0.0067 ± 0.0026	0.0426
*Intestinimonas*	0.0017 ± 0.0015	0.0065 ± 0.0037	0.0326
*Oscillibacter*	0.0012 ± 0.0013	0.0061 ± 0.0031	0.0019
*Veillonella*	0.0082 ± 0.0181	0.0001 ± 0.0001	0.0047
*Fibrobacter*	0.0000 ± 0.0000	0.0062 ± 0.0141	0.0164
*Parabacteroides*	0.0011 ± 0.0016	0.0053 ± 0.0033	0.0047
*Enterococcus*	0.0059 ± 0.0063	0.0000 ± 0.0000	0.0131
*Helicobacter*	0.0003 ± 0.0006	0.0040 ± 0.0032	0.0047
*Enterorhabdus*	0.0026 ± 0.0025	0.0002 ± 0.0002	0.0388
*Lachnoclostridium*	0.0019 ± 0.0021	0.0004 ± 0.0003	0.0388
*Oribacterium*	0.0000 ± 0.0001	0.0015 ± 0.0025	0.0047
*Eubacterium*	0.0015 ± 0.0017	0.0000 ± 0.0000	0.0124
*Parasutterella*	0.0009 ± 0.0011	0.0001 ± 0.0001	0.0388
*Papillibacter*	0.0001 ± 0.0001	0.0006 ± 0.0005	0.0016
*Anaerovibrio*	0.0000 ± 0.0000	0.0004 ± 0.0004	0.0187
*Anaerovorax*	0.0000 ± 0.0000	0.0004 ± 0.0004	0.0007
*Peptococcus*	0.0000 ± 0.0000	0.0001 ± 0.0002	0.0200
*Fusicatenibacter*	0.0001 ± 0.0002	0.0000 ± 0.0000	0.0027
*Flavonifractor*	0.0001 ± 0.0002	0.0000 ± 0.0000	0.0007
*Anaerofilum*	0.0000 ± 0.0000	0.0001 ± 0.0001	0.0124
*Gelria*	0.0000 ± 0.0000	0.0001 ± 0.0000	0.0080
*Barnesiella*	0.0000 ± 0.0000	0.0000 ± 0.0000	0.0019

SADI-S, single-anastomosis duodenal–ileal bypass with sleeve gastrectomy group; Sham-PF, sham operation with pair-feeding.

At the class level, SADI-S showed significantly higher abundances of Gammaproteobacteria, Erysipelotrichia, Verrucomicrobiae, Coriobacteriia, Actinobacteria, Chlamydia, and Betaproteobacteria and significantly lower abundances of Bacteroidia, Clostridia, Epsilonproteobacteria, Mollicutes, Fibrobacteria, and Opitutae. Gammaproteobacteria, belonging to the phylum Proteobacteria, showed the most significant change toward a higher abundance in the SADI-S group.

At the order level, Bacteroidales was the most abundant (46.4%), followed by Eubacteriales (20.6%) and Enterobacterales (9.2%). In the order Bacteroidales, 73.9% belonged to the family Prevotellaceae. Enterobacterales, belonging to the class Gammaproteobacteria of the phylum Proteobacteria, existed almost exclusively in the SADI-S group, with a proportion of 99.2%. SADI-S significantly changed the abundances of 12 orders compared to the sham-PF group: increased Enterobacterales, Erysipelotrichales, Verrucomicrobiales, Coriobacteriales, Bifidobacteriales, Chlamydiales, and Burkholderiales and decreased Bacteroidales, Clostridiales, Fibrobacterales, Campylobacterales, and Thermoanaerobacterales.

At the family level, Prevotellaceae had the highest proportion (34.3%), followed by Ruminococcaceae (9.6%) and Enterobacteriaceae (9.2%). SADI-S changed abundances of 21 families compared to the sham-PF group: increased Enterobacteriaceae, Erysipelotrichaceae, Verrucomicrobiaceae, Streptococcaceae, Coriobacteriaceae, Bifidobacteriaceae, Chlamydiaceae, Alcaligenaceae, Enterococcaceae, Clostridiaceae, and Eubacteriaceae and decreased Prevotellaceae, Ruminococcaceae, Lactobacillaceae, Bacteroidaceae, Rikenellaceae, Porphyromonadaceae, Fibrobacteraceae, Helicobacteraceae, Defluviitaleaceae, and Thermoanaerobacteraceae. Enterobacteriaceae of the phylum Proteobacteria existed almost exclusively in the SADI-S group, with a proportion of 99.2%.

At the genus level, SADI-S significantly changed the abundances of 33 bacteria such as the increase of anti-inflammatory bacteria (*Akkermansia* and *Bifidobacterium*) and the decrease of pro-inflammatory bacteria (*Bacteroides*). The increased *Bifidobacterium* also belonged to SCFA-producing bacteria.

### Relationships between the differential genera and the diabetic variables

**FIGURE 6:**
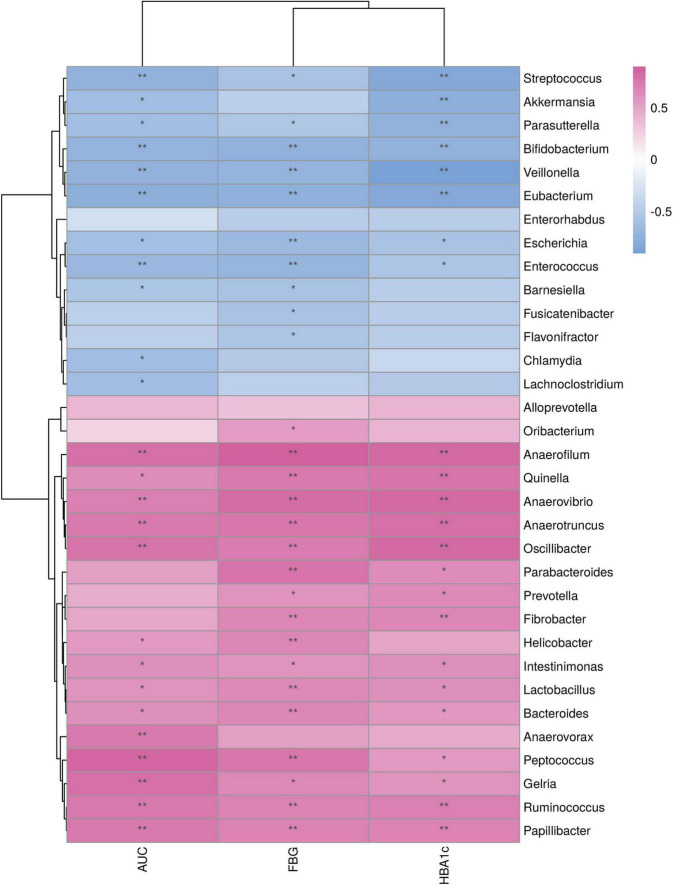
Relationship between the differential genera and metabolic variables. *p < 0.05; **p < 0.01. FBG, fasting blood glucose; AUC, area under the curve of the blood glucose level in intraperitoneal glucose tolerance test; HBA1c, glycated hemoglobin.

Relationships between the differential genera and the diabetic variables were assessed using Spearman’s rank correlation analysis (Figure 6). Diabetic parameters, including FBG and HBA1c levels and AUC of the glucose level in IPGTT, negatively correlated with abundances of *Streptococcus, Parasutterella, Bifidobacterium, Veillonella, Eubacterium, Escherichia*, and *Enterococcus* and positively correlated with abundances of *Anaerofilum, Quinella, Anaerovibrio, Anaerotruncus, Oscillibacter, Intestinimonas, Lactobacillus, Bacteroides, Peptococcus, Gelria, Ruminococcus*, and *Parabacteroides.*

## Discussion

To the best of our knowledge, this was the first study to investigate the effects of SADI-S on gut microbiota in T2D rats. In this study, SADI-S significantly decreased body weight and improved glucose metabolism in T2D rats, consistent with previous studies (Sánchez-Pernaute et al., 2015; Wu et al., 2022). Further, it significantly reduced the diversity and richness of gut microbiota, which indicated that the improvement effect of SADI-S on T2D may depend on the abundance of specific bacteria rather than the overall abundance and diversity of microbiota.

Effects of bariatric and metabolic surgery on the diversity and richness of gut microbiota have been previously evaluated. In some studies, SG significantly increased both diversity and richness of gut microbiota (Farin et al., 2020; Fukuda et al., 2022; Kural et al., 2022). Guo et al. (2017) reported that SG significantly increased the gut microbiota diversity but did not significantly change the richness. Lin et al. (2021) showed that SG did not significantly change both diversity or richness of gut microbiota. With the exception of one study (Shao et al., 2017), most showed that RYGB significantly increased the diversity (Guo et al., 2017; Murphy et al., 2017; Farin et al., 2020) and richness (Kong et al., 2013; Palleja et al., 2016; Farin et al., 2020) of gut microbiota. The present study demonstrated that SADI-S significantly reduced both diversity and richness of gut microbiota. Consistent with the present study, Mukorako et al. (2019) reported that BPD/DS significantly reduced the diversity and richness of the intestinal microbiota. SG is a restrictive bariatric procedure. In addition, the exclusion length of small intestine in BPD/DS and SADI-S is significantly longer than that of RYGB. Therefore, we speculate that the effects of bariatric and metabolic surgery on the diversity and richness of gut microbiota may depend on the exclusion length of small intestine. Of course, this viewpoint requires further research.

In this study, Bacteroidota was the most abundant phylum, and its level significantly decreased after SADI-S. Studies have reported a significantly increased abundance of Bacteroidota after RYGB and SG (Huang et al., 2014; Liu et al., 2018). It has also been reported that the abundance of Bacteroidota decreased after RYGB (Campisciano et al., 2018). In addition, some studies reported contrary findings concerning the effects of duodenojejunal bypass on the abundance of Bacteroidota. For example, Yu et al. (2020) reported that the abundance of Bacteroidota did not change significantly after single-anastomosis duodenojejunal bypass. However, Zhong et al. (2016) reported that the abundance of Bacteroidota in diabetic rats decreased significantly after duodenojejunal bypass. Study identified that the change in the abundance of Bacteroidota is related to whether diabetes is improved after surgery. Bacteroides, belonging to the phylum Bacteroidota, is a pro-inflammatory bacteria (Yan et al., 2022; Kang et al., 2023). In this study, the abundance of Bacteroides significantly decreased after SADI-S.

The abundance of Proteobacteria increased most significantly after SADI-S. Consistently, it increased significantly after duodenojejunal bypass and RYGB (Zhong et al., 2016; Liu et al., 2018; Yu et al., 2020), concomitant with the significant improvement of T2D. The results from Liu et al. (2018) showed that gastric bypass decreased the abundance of Verrucomicrobiota despite lacking of significant difference. In contrast, our study demonstrated that SADI-S significantly increased the abundance of the phylum Verrucomicrobiota. *Akkermansia*, belonging to the phylum Verrucomicrobia, is an anti-inflammatory bacteria (Luo et al., 2022; Del Chierico et al., 2023), and its abundance is negatively correlated with the level of insulin resistance (Dao et al., 2016; Plovier et al., 2017; Konturek et al., 2018). We also found that the abundance of *Akkermansia* was significantly increased after SADI-S, concomitant with the significant improvement of T2D. In the present study, SADI-S also significantly changed the abundance of Actinobacteria, Tenericutes, and Fibrobacteres. Previous studies have demonstrated that the abundance of the phylum Actinomycetota in diabetic rats significantly increased after RYGB (Liu et al., 2018) and single-anastomosis duodenojejunal bypass (Yu et al., 2020), consistent with our findings. The abundance of the family Coriobacteriaceae, belonging to the phylum Actinomycetota, decreased significantly in patients with diabetes compared to the healthy population (Liu et al., 2018). In the present study, SADI-S significantly decreased the blood glucose level and increased the abundance of Coriobacteriaceae in diabetic rats. Thus, Coriobacteriaceae may play a vital role in bariatric surgery regulating host glucose homeostasis. *Bifidobacterium*, belonging to the phylum Actinomycetota, is also an anti-inflammatory bacteria (Luo et al., 2022; Del Chierico et al., 2023). The abundance of *Bifidobacterium* decreases in patients with T2D and increases with BPD/DS and duodenal jejunal bypass (Mukorako et al., 2019; Yu et al., 2020), consistent with our findings. Low-grade chronic inflammation is one of the pathogenesis of T2D. Therefore, the increase of the above-mentioned anti-inflammatory bacteria (*Akkermansia* and *Bifidobacterium*), and the decrease of the pro-inflammatory bacteria (*Bacteroides*) may be the mechanism by which SADI-S improves T2D.

Fecal metabolites are the products of complex metabolic activities of gut microbiota in the body, and also serve as a bridge for the interaction between the host and intestinal microorganisms. Our previous study demonstrated that the level of fecal SCFA in T2D rats significantly increased after SADI-S (Wang et al., 2022). SCFA can stimulate ileocolic L cells to secrete GLP-1, promoting insulin secretion and islet β-cell proliferation. It has also been shown (Roy et al., 2020) that SCFA can not only block the expression and secretion of pro-inflammatory cytokines, but also improve insulin signaling by reversing IRS-1 serine phosphorylation. In the present study, the abundance of *Bifidobacterium*, a kind of SCFA-producing bacteria in T2D rats significantly increased after SADI-S. BCAA are closely related to the development of T2D, and their levels are significantly elevated in patients with T2D (Wang et al., 2011, 2017; Zheng et al., 2016). BCAA levels were significantly reduced in patients with T2D after bariatric surgery (Arora et al., 2015; Luo et al., 2016). Previous study (Wang et al., 2022) found that the elevated levels of BCAA are associated with the increased abundance of bacteria that synthesize BCAA such as *Prevotella* (Pedersen et al., 2016). Consistent with the above findings, our previous (Wang et al., 2022) and present study found that the levels of BCAA and the abundance of *Prevotella* in T2D rats significantly decreased after SADI-S.

The clinical applications based on the above-mentioned results are as follows: firstly, we may improve glucose metabolism in T2D patients by reducing their inflammation levels. For example, transplanting intestinal anti-inflammatory bacteria (Akkermansia and Bifidobacterium) may reduce the body’s inflammation level and thereby improve glucose metabolism in T2D patients. Secondly, for those T2D patients who have undergone bariatric surgery, we should advise them to reduce their BCAA intake to reduce the risk of T2D recurrence. Thirdly, T2D patients should be encouraged to increase dietary fiber intake because it can enrich SCFA-producing bacteria, improve the level of intestinal SCFA, activate intestinal cells to secrete GLP-1, and increase insulin levels in patients. In our future studies, we will reversely verify the hypoglycemic effect of SADI-S through fecal microbiota transplantation. Firstly, we will collect the gut microbiota of T2D rats treated with SADI-S surgery and then transplant them into T2D rats so as to observe whether the gut microbiota of T2D rats after SADI-S intervention has a hypoglycemic effect. If it is effective, we will perform SADI-S surgery on T2D patients. After their T2D is completely relieved, we will transplant their gut microbiota to T2D patients so as to observe whether the gut microbiota of T2D patients after SADI-S intervention has a hypoglycemic effect.

Although this was the first study to explore the effects of SADI-S on gut microbiota in T2D rats, it had some limitations. First, other bariatric surgeries, such as RYGB or SG, were not set as the control group of SADI-S. Therefore, studies comparing the effects of various types of bariatric surgeries (SADI-S, RYGB, and SG) on gut microbiota should be performed in the future. Second, a significant number of rats died from the high operational difficulty of SADI-S surgery, resulting in a relatively smaller sample size in the SADI-S group. Third, we did not further validate the effects of the differential bacteria caused by SADI-S surgery on glucose metabolism in T2D rats.

## Conclusion

Single-anastomosis duodenal–ileal bypass with sleeve gastrectomy significantly improved glucose metabolism and decreased the species richness and diversity of gut microbiota. SADI-S significantly altered the gut microbiota composition of T2D rats, including increased anti-inflammatory bacteria (*Akkermansia* and *Bifidobacterium*) and decreased pro-inflammatory bacteria (Bacteroides). The blood glucose level of rats was positively correlated with the abundances of 12 bacteria, including *Bacteroides*, but negatively correlated with the relative abundance of seven bacteria, including *Bifidobacterium*. Alternations in gut microbiota may be the mechanism through which SADI-S improves T2D. More studies are required in the future to validate these effects.

## Data availability statement

The data presented in the study are deposited in the NCBI repository, accession number PRJNA1111458.

## Ethics statement

The animal study was approved by the Animal Experiment Ethics Committee of the First Hospital of Jilin University. The study was conducted in accordance with the local legislation and institutional requirements.

## Author contributions

LW: Conceptualization, Data curation, Formal analysis, Methodology, Writing – original draft. SL: Data curation, Software, Writing – review & editing. TJ: Conceptualization, Funding acquisition, Methodology, Validation, Writing – review & editing.

## Footnotes

1
http://drive5.com/uparse/

## Supplementary material

Supplementary Figure 1 The Venn plot of OTU for different groups.

Supplementary Figure 2 The β-diversity analysis of gut microbiota by non-metric multidimensional scaling (NMDS).

Supplementary Figure 3 The cladogram of plots presented the LEfSe results of the biological structure of gut microbiota. Gut bacteria marked with small circles highlight significant differences of relative abundance between the two groups.
