# Supplementary figures and images for: Effects of single-anastomosis duodenal–ileal bypass with sleeve gastrectomy on gut microbiota and glucose metabolism in rats with type 2 diabetes
Source: Front Microbiol. 2024 May 27;15:1357749. doi: 10.3389/fmicb.2024.1357749 (PMC11165999; doi:10.3389/fmicb.2024.1357749)

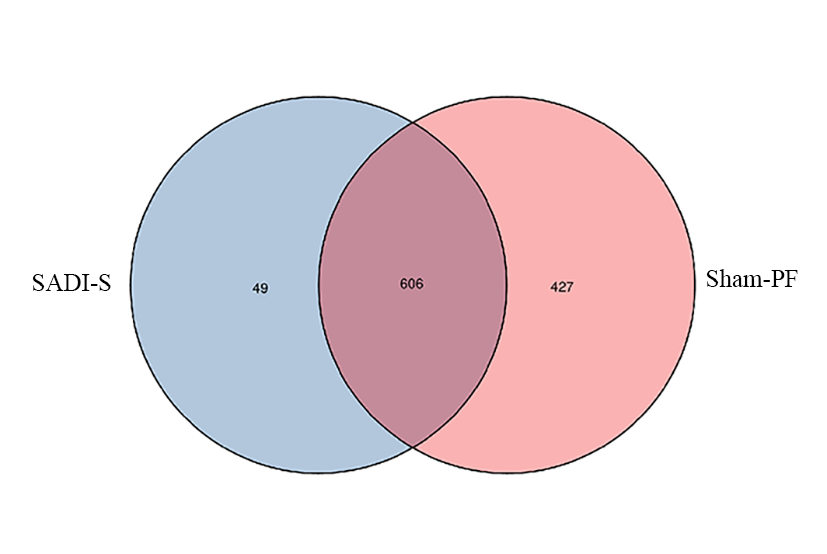

Supplement: Supplementary Figure 1 — The Venn plot of OTU for different groups. [file Image_1.TIF]

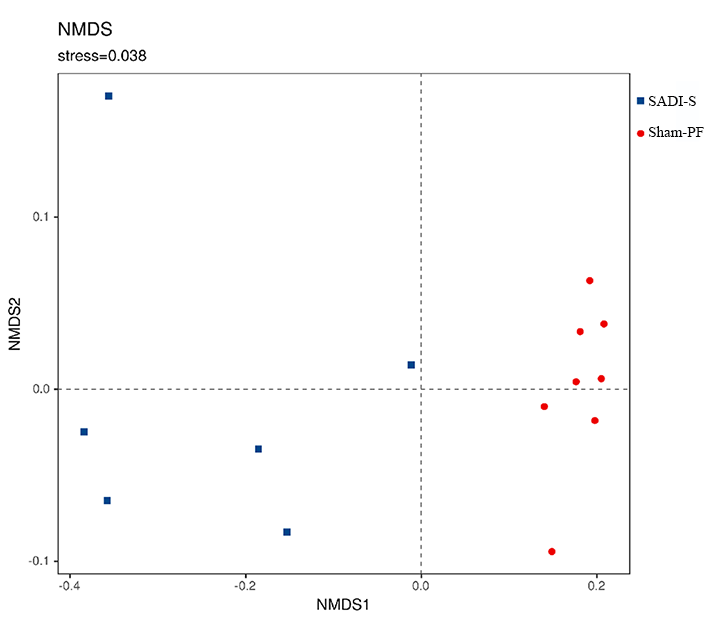

Supplement: Supplementary Figure 2 — The β-diversity analysis of gut microbiota by non-metric multidimensional scaling (NMDS). [file Image_2.TIF]

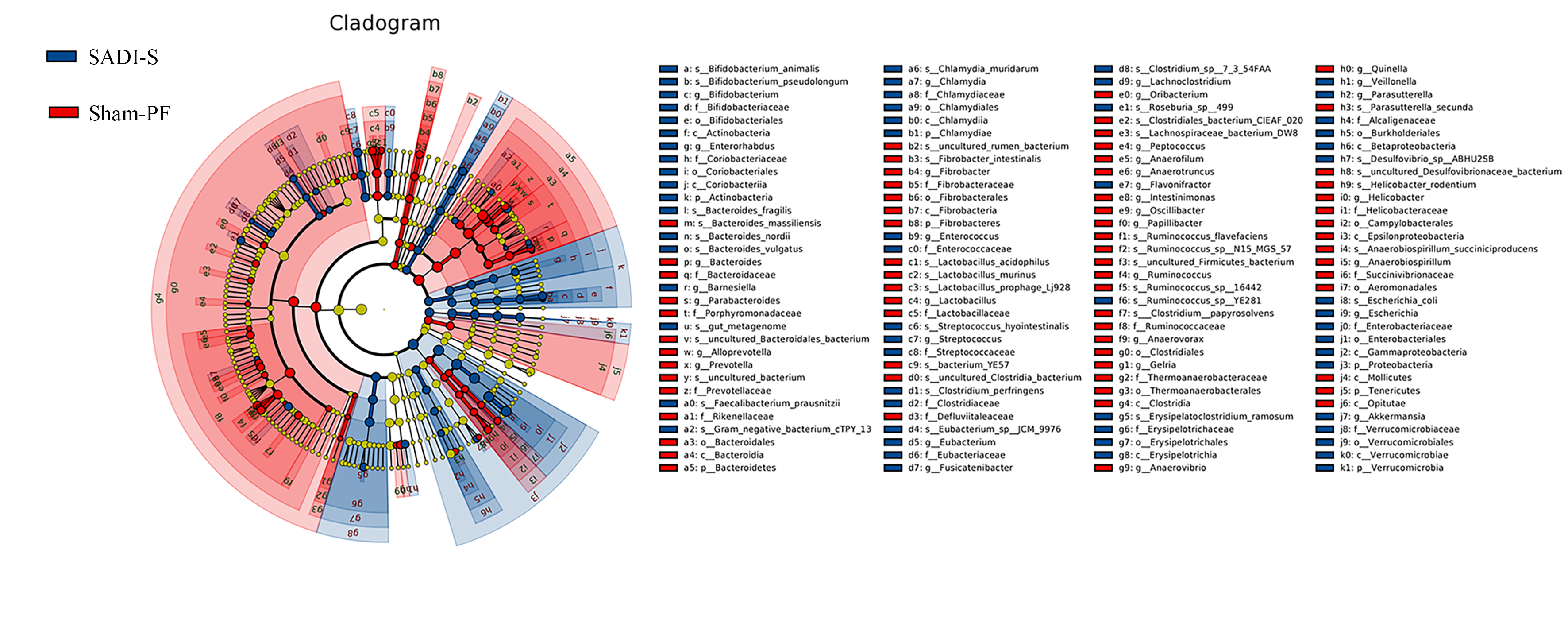

Supplement: Supplementary Figure 3 — The cladogram of plots presented the LEfSe results of the biological structure of gut microbiota. Gut bacteria marked with small circles highlight significant differences of relative abundance between the two groups. [file Image_3.TIF]
